# Supplementary figures and images for: Increased Expression of Interferon-Induced Transmembrane 3 (IFITM3) in Stroke and Other Inflammatory Conditions in the Brain
Source: Int J Mol Sci. 2022 Aug 10;23(16):8885. doi: 10.3390/ijms23168885 (PMC9408431; doi:10.3390/ijms23168885)

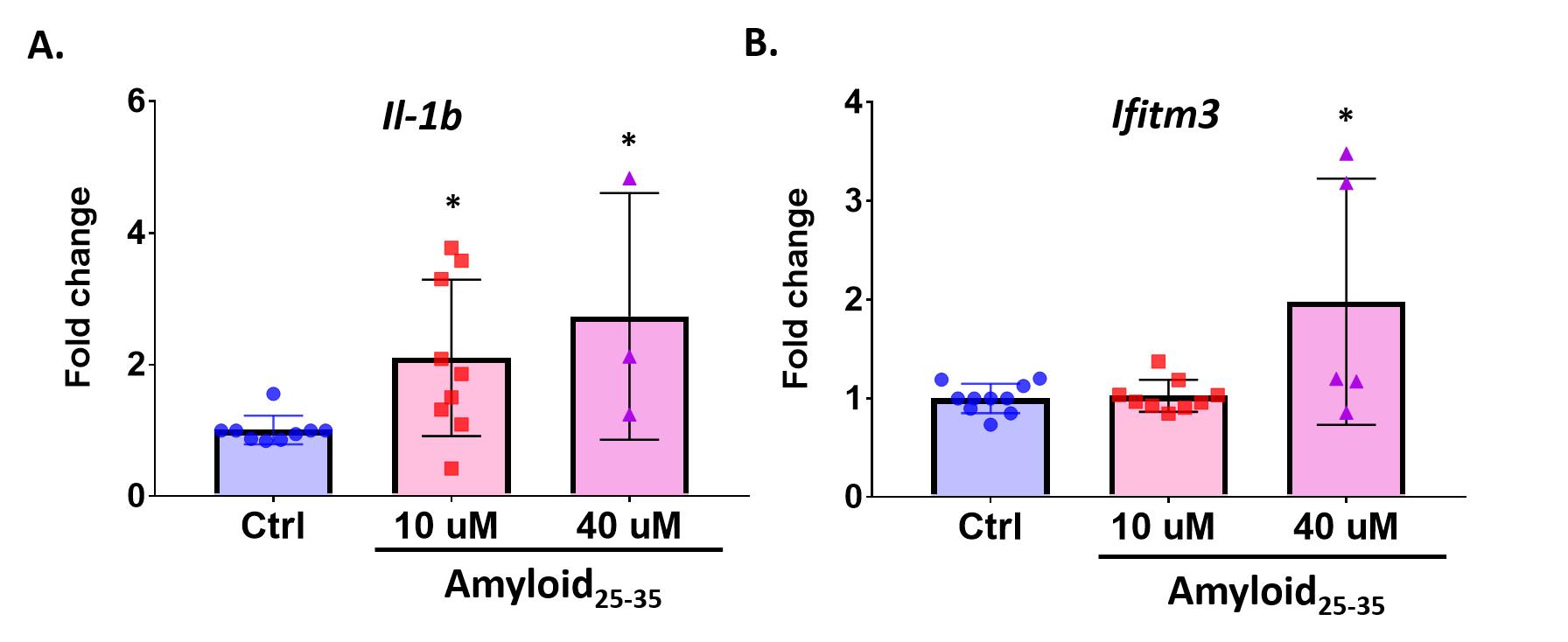

Supplement: Supplementary file 1 [file ijms-23-08885-s001.zip › ijms-1854564-supplementary-Figure S1.jpg]
